# Tumor Response to Immune-Oncology Therapy Despite High-Dose Steroid Intervention in a Patient With Papillary Renal Cell Carcinoma and Immune-Related Myasthenia Gravis

**DOI:** 10.7759/cureus.80136

**Published:** 2025-03-06

**Authors:** Liyan Mazahreh, Farah Mazahreh, Bayan Alqtishat, Mazin Safar

**Affiliations:** 1 Internal Medicine, University of Arkansas for Medical Sciences, Little Rock, USA; 2 Neurology, University of Arkansas for Medical Sciences, Little Rock, USA; 3 Hematology and Medical Oncology, University of Arkansas for Medical Sciences, Little Rock, USA

**Keywords:** high-dose corticosteroids, immune-checkpoint inhibitors, immune mediated myasthenia gravis, immune-related adverse effects, papillary renal cell cancer, tumor response

## Abstract

Immune-oncology (IO) therapies, particularly immune checkpoint inhibitors, have significantly altered the treatment landscape for various cancers, including renal cell carcinoma (RCC). Unfortunately, immune-related adverse events (irAEs) during therapy often necessitate corticosteroid intervention, which may theoretically impair the anti-tumor immune response. In this article, we describe a unique case in which a patient with advanced papillary RCC developed immune-related myasthenia gravis (MG) while on nivolumab (a PD-1 inhibitor) and cabozantinib. Even while giving corticosteroid treatment for myasthenia gravis, the patient exhibited a striking treatment response, with substantial tumor shrinkage and regression of regional lymphadenopathy. This case highlights that IO agents can remain efficacious even in the presence of significant immunosuppression, raising important mechanistic and clinical considerations for managing irAEs.

## Introduction

Renal cell carcinoma (RCC), particularly the papillary variant, often presents with locally advanced or metastatic disease [1]. Treatment options for advanced RCC include immune checkpoint inhibitors (ICIs), which target inhibitory receptors such as programmed death-1 (PD-1) or cytotoxic T-lymphocyte-associated protein 4 (CTLA-4) to enhance T-cell-mediated anti-tumor activity [2]. Unfortunately, these agents are associated with a broad spectrum of immune-related adverse events (irAEs), which may require high-dose steroids to control inflammation [3,4].

Corticosteroids, while essential for treating severe irAEs, can suppress T-cell function and other immune pathways, theoretically reducing the anti-tumor efficacy of ICIs [5]. Nonetheless, there is an ongoing debate regarding the extent to which steroids truly diminish the benefits of immunotherapy. This debate is particularly relevant in scenarios where a patient experiences a robust tumor response despite concurrent high-dose steroid use [6,7].

Myasthenia gravis (MG) is an example of a severe irAE characterized by autoantibody- and T-cell-driven neuromuscular dysfunction. It often presents with ocular symptoms (ptosis, diplopia) but can progress to generalized weakness [8,9]. In rare instances, MG arises de novo in patients on ICIs or worsens in those with a predisposition. Importantly, overlapping myositis or elevated creatine kinase (CK) can sometimes occur, highlighting the need for vigilance [10-12].

This case is notable for demonstrating substantial tumor regression in a patient with advanced papillary RCC who received nivolumab and cabozantinib but required intensive immunosuppression for immune-related MG. We discuss the paradox of maintaining anti-tumor immunity under these circumstances and highlight mechanistic considerations that could explain such an outcome, along with potential confounding factors such as underlying chronic kidney disease (CKD) and the role of cabozantinib [6].

## Case presentation

**Figure 1:**
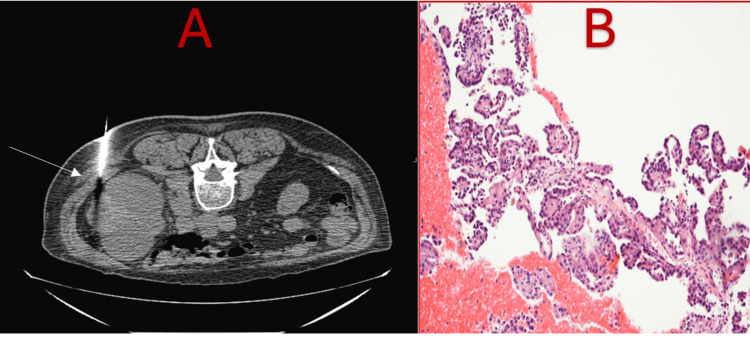
Core needle biopsy of the right kidney mass with representative pathology. Core needle biopsy of the right kidney mass (A) with representative histologic appearance of the tumor H&E stain revealing papillary projections with fibrovascular cores (B).

An 85-year-old male with a medical history significant for atrial fibrillation, CKD stage 4, and benign prostatic hyperplasia presented with gross hematuria. A CT scan of the chest, abdomen, and pelvis revealed a large right renal mass, regional lymphadenopathy, and possible mediastinal lymph node involvement. A CT-guided biopsy confirmed papillary RCC (Figure 1). Given the advanced stage and inoperability, the patient was referred for systemic therapy.

The patient was started on nivolumab (240 mg every two weeks) and cabozantinib (20 mg daily) on November 30, 2023. After four weeks, liver function tests (LFTs) showed mildly elevated aspartate transaminase (AST) (50 U/L; reference range: 10-40 U/L) and alanine transaminase (ALT) (60 U/L; reference range: 7-56 U/L). The rationale for starting steroids at this point was twofold: (1) a concern for emerging hepatic irAE and (2) a more pronounced LFT elevation on recheck. Consequently, prednisone 0.5 mg/kg/day was initiated, and ICI therapy was held temporarily.

Shortly thereafter, the patient developed rapidly progressive ptosis requiring manual eyelid elevation. Although he had no prior generalized fatigue or proximal muscle weakness, neurological evaluation raised suspicion of ICI-induced MG, potentially with an element of subclinical myositis. Serum CK and anti-striated muscle antibodies were not obtained at that time due to logistical constraints and the patient’s comorbidities. Anti-acetylcholine receptor (AChR) antibodies and anti-MuSK antibodies were also not measured because of rapid clinical progression and the decision to initiate empiric therapy. No swallowing or respiratory deficits emerged, but the ocular symptoms developed rapidly faster than typical idiopathic MG.

The patient was admitted for high-dose intravenous (IV) methylprednisolone (1 g/day for five days), followed by oral prednisone at 1 mg/kg/day, along with pyridostigmine and plasmapheresis. Plasmapheresis was performed a total of five times between January 10 and January, 15, 2024. IVIG was avoided due to his CKD. Over several weeks, his ocular symptoms markedly improved.

Immunologic labs (e.g., T-cell subsets, flow cytometry, or mitogen studies) were not performed due to his advanced age, comorbidities, and the acute need for therapeutic interventions. Hence, we cannot fully assess whether T-cell numbers remained stable or whether T-cell function was preserved despite steroid therapy.

**Figure 2:**
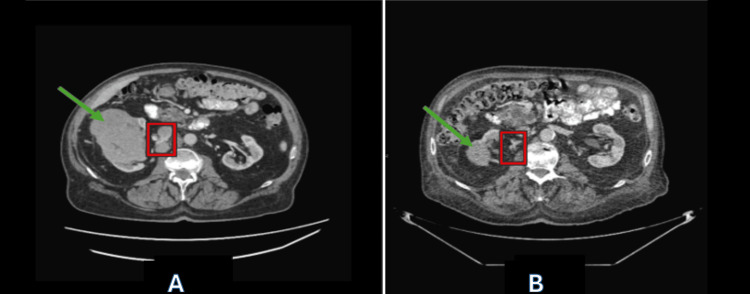
Pre- and post-treatment CT scan revealing significant regression of kidney mass and lymph node. CT scan pre-treatment (A) and post-treatment (B) showing substantial regression of the kidney mass (green arrow) and lymph node (red box).

Despite this degree of immunosuppression, a follow-up CT scan on April 16, 2024, (approximately four months into therapy) showed significant tumor response. The right renal mass shrank from 10.6 × 7.0 cm to 3.8 × 3.4 cm (solid component) and from 10.8 × 6.9 cm to 4.8 × 3.4 cm (cystic component). The largest aortocaval lymph node shrank from 2.1 cm to 6 mm, and the right hilar lymph node decreased from 11 mm to 6 mm with no new metastases (Figure 2). This shrinkage continued concurrently with steroid therapy, suggesting that robust immune activity was established before, or persisted despite, corticosteroid administration.

The patient’s taper involved gradually reducing prednisone by approximately 10 mg every two weeks if symptoms remained controlled. By the time of imaging on April 16 16, 2024, he was on prednisone 20 mg daily with stable liver function and resolving MG. Future renal cancer management currently includes cautious continuation of cabozantinib and close monitoring [6]. Re-challenge with nivolumab may be considered if the patient’s MG remains stable and the net benefit/risk is deemed acceptable by a multidisciplinary team [2,11].

To summarize the key clinical events and steroid taper, we present the timeline in Table 1.

**Table 1:** Timeline of key clinical events and steroid taper LFT, liver function test; ICI, immune checkpoint inhibitor; MG, myasthenia gravis

Date	Event
November 30, 2023	Initiated nivolumab (240 mg once every 2 weeks) + cabozantinib (20 mg daily)
December 30, 2023	First LFT elevation noted → prednisone 0.5 mg/kg/day started; ICI therapy held
January 10, 2024	Rapid onset of ocular MG symptoms → admission for MG
January 10, to January 15, 2024	High-dose IV methylprednisolone (1 g/day for 5 days) + plasmapheresis (5 sessions total)
January 16, 2024	Switched to oral prednisone 1 mg/kg/day; continued pyridostigmine
February 1, 2024	Began prednisone taper (reducing by ~10 mg every 2 weeks)
April 16, 2024	Follow-up imaging: significant tumor and lymph node regression

## Discussion

This case underscores a paradox in immuno-oncology: robust anti-tumor activity can persist despite high-dose steroid use. ICIs enhance T-cell function by blocking inhibitory receptors (e.g., PD-1), while steroids broadly downregulate immune responses, including T-cell proliferation and cytokine production [5,9]. Typically, clinicians worry that concurrent steroid therapy negates the benefits of ICIs. However, growing evidence suggests that once an effective anti-tumor immune response is established, short-term or carefully managed steroid use may not completely abolish it [5,9]. It is possible that T cells remain functionally active, at least to some extent, despite reduced numbers.

Additionally, MG is a recognized irAE of checkpoint inhibitor therapy. Though it can be severe, particularly in older patients or those with CKD, it often responds well to high-dose steroids, plasmapheresis, and supportive measures such as pyridostigmine [4,12]. Our patient’s rapid improvement emphasizes the importance of early recognition and aggressive management. Repetitive nerve studies or single-fiber EMG could have confirmed the diagnosis in a more classic manner, but the urgent clinical scenario prioritized immediate therapy over extensive electrophysiological testing. The lack of anti-AChR or anti-MuSK antibody titers is also a limitation but reflects real-world constraints.

The timing of immune response induction appears crucial. Some hypothesize that a robust T-cell activation phase, achieved before introducing steroids, can drive ongoing tumor control even when immunosuppression is introduced later [10]. Other immune effectors, such as NK cells or macrophages, may exhibit reduced sensitivity to steroids, continuing to orchestrate tumor cell clearance [10]. Meanwhile, cabozantinib exerts antiangiogenic and immunomodulatory effects [6], which might further support tumor shrinkage, representing a potential confounding factor in interpreting the pure effect of ICIs.

Potential mechanistic insights

The observed tumor response despite high-dose steroid intervention may be attributed to several factors. Differential steroid sensitivity suggests that while T cells might be more vulnerable to corticosteroids, other immune cells such as natural killer (NK) cells or macrophages could retain partial activity, allowing continued anti-tumor effects [10]. Additionally, pre-established immunity may allow tumor-directed T-cell clones that have already expanded to remain functionally active despite short-term steroid administration, thereby maintaining their cytotoxic functions [9]. Furthermore, combination effects may also play a role, as cabozantinib’s inhibition of VEGF receptors and other kinases could complement the immune response by modifying the tumor microenvironment, possibly enhancing anti-tumor efficacy [6].

Limitations and future research

This case report is based on a single-patient observation, necessitating caution in generalizing the findings. The lack of immune analysis is another limitation, as detailed assessments of T-cell subsets or functional assays (e.g., mitogen tests) were unavailable, leaving unanswered questions regarding the potency of immune effectors in the presence of corticosteroids [5]. Future research should explore the optimal timing of steroid initiation relative to immune checkpoint inhibitor therapy, which immune cell populations are relatively resistant to steroid-mediated suppression, and biomarkers that predict tumor response despite immunosuppression [9,10].

## Conclusions

This case demonstrates that immuno-oncology therapies, namely nivolumab and cabozantinib, can produce substantial tumor regression despite intensive steroid treatment for an irAE (MG). The observed paradox, effective immunosuppression alongside continued anti-tumor activity, suggests that factors such as timing, cellular targets of immunosuppression, and individual patient immune profiles may modulate ICI efficacy. While caution is warranted when extrapolating from a single case, these findings challenge the assumption that steroids categorically negate ICIs. Mechanistic studies on steroid-tolerant immune subsets and the impact of combination regimens (such as cabozantinib + nivolumab) are needed to optimize treatment strategies. For our patient, continuing cabozantinib with close monitoring is planned, and re-challenging with nivolumab will be considered if his MG remains controlled. Further research will help define best practices for balancing irAE management with sustained oncologic benefit.
